# Primary Stability of Zirconia Dental Implants with Cylindrical and Tapered Designs Across Varying Bone Densities: An In Vitro Evaluation

**DOI:** 10.3390/dj12110356

**Published:** 2024-11-05

**Authors:** Diogo Fernandes, Francisco Cavaco, Filipe Freitas, Duarte Marques, João Caramês, André Moreira

**Affiliations:** 1 Faculdade de Medicina Dentária da Universidade de Lisboa, 1600-277 Lisbon, Portugalfrancisco.cavaco@edu.ulisboa.pt (F.C.); 2 Department of Oral Surgery and Oral Medicine, Faculdade de Medicina Dentária da Universidade de Lisboa, 1600-277 Lisbon, Portugal; 3 Oral Biology and Biochemistry Research Group, Faculdade de Medicina Dentária da Universidade de Lisboa, 1600-277 Lisbon, Portugal

**Keywords:** dental implants, zirconium, bone density, resonance frequency analysis

## Abstract

**Background:** While titanium implants are widely recognized for their clinical success, zirconia implants have emerged as a metal-free alternative. This study aimed to evaluate the influence of zirconia implant macrogeometry and bone density on primary implant stability. **Methods:** Two types of zirconia implants were tested—the Neodent^®^ Zi Ceramic Implant and the Straumann^®^ PURE Ceramic Implant, that were placed into polyurethane foam blocks mimicking different bone densities (10 PCF, 15 PCF, 20 PCF, 30 PCF, and 40 PCF). Each implant type was inserted and removed multiple times, with primary stability measured using resonance frequency analysis via the Osstell^®^ Beacon device. Statistical tests, including the Shapiro–Wilk test, *t*-tests, the Mann–Whitney U test, and the Kruskal–Wallis test, were applied, with significance set at 5% (*p* < 0.05). **Results:** The tapered Neodent^®^ Zi Ceramic Implant consistently showed higher ISQ values across all foam densities compared to the Straumann^®^ PURE Ceramic Implant (*p* = 0.035). Additionally, lower-density foams exhibited lower stability scores (*p* < 0.05). **Conclusion:** The study concludes that both the macrogeometry of zirconia implants and bone density significantly affect primary implant stability. Specifically, tapered implants demonstrated higher stability than cylindrical designs, suggesting that implant macrogeometry and bone density should be considered for optimal primary stability in clinical settings.

## 1. Introduction

For many years, titanium has been extensively documented as a biomaterial and is widely considered the “gold standard” in implantology [1]. Research has demonstrated survival rates exceeding 95% after a decade [1,2,3,4].

However, as studies on titanium implants have proliferated, several drawbacks have been identified, including aesthetic concerns in anterior regions and areas with a thin gingival biotype (less than 2 mm thick); allergic reactions [1,5,6], particularly type IV immunological responses [7,8], which can lead to the sensitization and proliferation of reactive T lymphocytes, triggering an inflammatory cascade that results in clinical symptoms and a potential loss of peri-implant tissue [8]; the presence of titanium particles in surrounding soft and hard tissues, as well as regional lymph nodes [5,6,9]; and titanium particles and ions being capable of provoking the release of pro-inflammatory cytokines involved in bone resorption, such as TNF-α, IL-1β, and RANK-L, which has recently been associated with the development of peri-implantitis [10].

In contrast, zirconia has emerged as a versatile and promising alternative, thanks to its excellent mechanical properties, high biocompatibility, and superior aesthetic results [6,8,9,11,12], particularly in aesthetic zones like the anterior mandible, where a thin, soft tissue biotype is common [13]. Zirconia abutments provide a more suitable color match between the peri-implant mucosa and natural teeth. Additionally, zirconia implants have shown average scores for the Pink Esthetic Score, White Esthetic Score, and Peri-Implant and Crown Index that exceed the clinical acceptability threshold [14].

Unlike titanium implants, zirconia does not cause local or systemic toxic effects, chronic inflammation, the release of pro-inflammatory cytokines, or arachidonic acid metabolism [15]. This makes it the preferred choice for patients with metal hypersensitivity, especially those allergic to titanium implants [12,16]. Zirconia has low surface energy, resulting in a reduced affinity for the adhesion and colonization of periodontal pathogens compared to titanium [1,11,12,13,16,17] and a lower prevalence of peri-mucositis [1]. The BIC (Bone-to-Implant Contact) values of zirconia implants do not show statistically significant differences compared to titanium implants. Moreover, implants with a modified surface may have the potential to reduce early implant failure [6]. Meta-analyses have shown that zirconia implants have survival rates comparable to those of titanium implants at 1 and 2 years, with rates of 98.3% and 97.2%, respectively, and a mean marginal bone loss of 0.7 mm at 1 year [18]. Furthermore, prospective studies have reported survival rates of 97.5% [19] and 98.5% [20] at 3 years, and 98.4% at 5 years [21].

Osseointegration is a dynamic process during which primary stability is gradually replaced by secondary stability. After implant placement, the fixation of the implant is ensured by primary stability. This mechanical fixation corresponds to the direct contact between the surface of the implant and the walls of the bone bed and is influenced by factors such as the implant’s macrogeometry and microgeometry, the quality of the host bone, the type of bone bed preparation (press-fit), and the vertical position of the implant in relation to the bone crest.

On the other hand, secondary stability corresponds to a biological connection, characterized by bone regeneration and remodeling, which begins with the initial deposition of new bone on the implant surface [22].

The design of the implant is crucial for achieving primary and secondary stability for long-term osseointegration and implant success. Various macrogeometric factors can affect stability, including implant shape, diameter, length, threads, and surface roughness [23]. Conical implants seem to offer enhanced primary stability through bone condensation in areas of reduced bone quality, producing a certain lateral and vertical compressive force in the surrounding cortical bone, leading to a more uniform distribution of forces [23,24]. Furthermore, they are an alternative to prevent the perforation of vestibular and lingual bone walls in areas with concavities, especially in situations where the alveolar crest is thin, creating more favorable conditions for the safe placement of implants between adjacent roots [24,25].

Implant stability is a decisive variable at different stages of rehabilitation, as it is useful for predicting early implant failure, assessing the quality of osseointegration, and determining whether immediate loading is feasible [26]. Resonance frequency analysis (RFA) is a simple, practical, objective, and reproducible diagnostic method that allows for reliable and repeatable measurements of implant stability [27]. This technique is based on the emission of resonance frequencies from 3000 to 8500 Hz, allowing for the evaluation of implant stability and osseointegration, regardless of the timing of the stability measurement [27,28]. The most current and widely used version by the scientific community is Osstell^®^, a non-invasive, compact, and wireless device [26] that uses a magnetic transducer (Smartpeg™) screwed onto the implant. When activated by magnetic impulses emitted by the Osstell^®^ probe, the transducer vibrates, creating a resonance frequency captured and expressed as an Implant Stability Quotient (ISQ), ranging from 0 to 100 [28]. With the latest device, Osstell^®^ Beacon, only two measurements are taken in different directions, mesio-distal and bucco-lingual, with the device’s tip 2–4 mm from the top of the SmartPeg™ (without touching it) at an angle of approximately 45 degrees [29].

When ISQ values exceed 70, high implant stability is indicated; values between 60 and 69 indicate medium stability; and values below 60 are considered to indicate low implant stability [30]. Although various factors are known to influence primary stability, the most reliable method for in vivo measurement and quantification is currently the RFA measurement of the implant [26].

The aim of this in vitro study was to compare the primary stability of two zirconia implants with different macrogeometries using RFA and to clarify the impact of bone density on primary stability. This research is particularly relevant as zirconia is gaining popularity as an alternative to titanium; yet, the scientific evidence supporting its use remains limited. Additionally, differences in macrogeometry and the mechanical properties of zirconia influence primary stability, a key factor for the long-term success of implants. Examining the influence of macrogeometry and bone density is crucial, as both factors play a significant role in achieving optimal primary stability, especially in areas with varying bone quality.

To address these issues, the study is guided by the following hypotheses: For macrogeometry, the null hypothesis (H_0_) is that there are no significant differences between primary stability values and the different geometries of the implants, while the alternative hypothesis (H_1_) posits that there are significant differences between these values. For bone density, the null hypothesis (H_0_) is that there are no significant differences between primary stability results when comparing different bone densities, whereas the alternative hypothesis (H_1_) suggests that there are significant differences between these results.

Testing these hypotheses will help elucidate the roles of implant macrogeometry and bone density in achieving optimal primary stability, providing valuable insights into the use of zirconia implants in clinical practice.

## 2. Materials and Methods

### 2.1. Implants and Polyurethane Blocks

**Figure 1:**
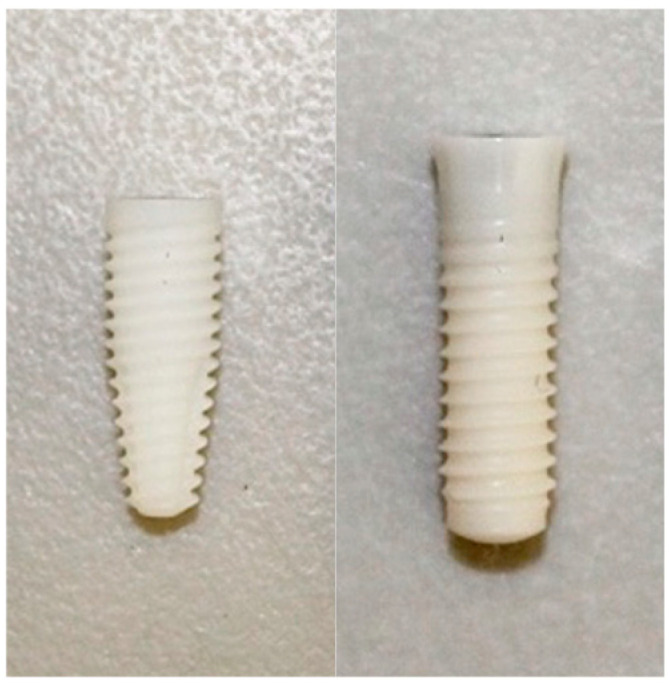
Neodent^®^ Zi Ceramic Implant (Neodent, Curitiba, Brazil) and Straumann^®^ Pure Ceramic Implant (Institut Straumann AB, Basel, Switzerland).

Two zirconia implants from different manufacturers and specifications were used (Figure 1): the Neodent^®^ Zi Ceramic Implant (Neodent, Curitiba, Brazil) is a Y-TZP zirconia implant characterized by a conical design with a double trapezoidal thread profile. It has an endosteal diameter of 4.3 mm and a length of 11.5 mm. The implant is white in color and features a ZiLock^®^ connection (Neodent, Curitiba, Brazil). The surface treatment applied is Neoporos^®^. The Straumann^®^ Pure Ceramic Implant (Institut Straumann AB, Basel, Switzerland) is also a Y-TZP zirconia implant, designed with a cylindrical, two-piece, tissue-level configuration. This implant has an endosteal diameter of 4.1 mm and a length of 12 mm. It is ivory in color and includes an internal regular diameter connection. The surface treatment used is ZLA^®^ (Institut Straumann AB, Basel, Switzerland).

**Figure 2:**
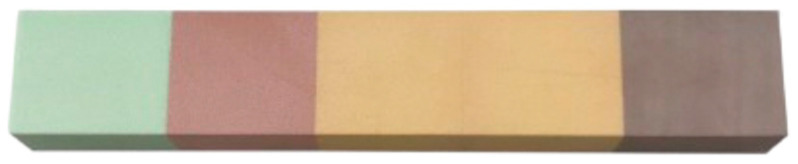
National Ossos^®^ polyurethane foam block.

Only one polyurethane foam block of 5 densities, without cortical bone, from the brand Nacional Ossos^®^ reference 12458 (Nacional Ossos, Jaú, São Paulo, Brasil) was used, with overall dimensions of 225 × 45 × 25 mm (Figure 2). Each density section had the following dimensions: 45 × 45 × 25 mm.

▪ Block 1: Density of 10 PCF (0.16 g/cm^3^) and light-green color.
▪ Block 2: Density of 15 PCF (0.24 g/cm^3^) and pink color.
▪ Block 3: Density of 20 PCF (0.32 g/cm^3^) and light-orange color.
▪ Block 4: Density of 30 PCF (0.48 g/cm^3^) and dark-orange color.
▪ Block 5: Density of 40 PCF (0.96 g/cm^3^) and brown color.

According to the brand, the five densities correspond to the following bone types: 40 PCF—type I; 30 PCF—type II; 20 PCF—between types II and III; 15 PCF—type III; 10 PCF—type IV.

### 2.2. Sample Description

The sample for this study consisted of n = 90 placements, including 50 placements of the Straumann^®^ Pure Ceramic Implant (Institut Straumann AG, Basel, Switzerland) and 40 placements of the Neodent^®^ Zi Ceramic Implant (Neodent, Curitiba, Brazil), as the brand differentiates protocol specifically for the four bone types I, II, III, and IV. Consequently, the implantation of this implant was not performed at the density of 20 PCF.

### 2.3. Drilling and Insertion Protocols

**Figure 3:**
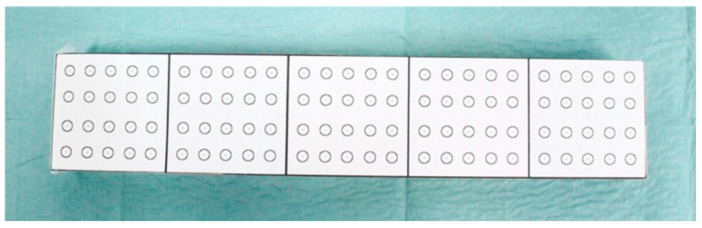
Paper template with the election points for performing the osteotomy.

**Figure 4:**
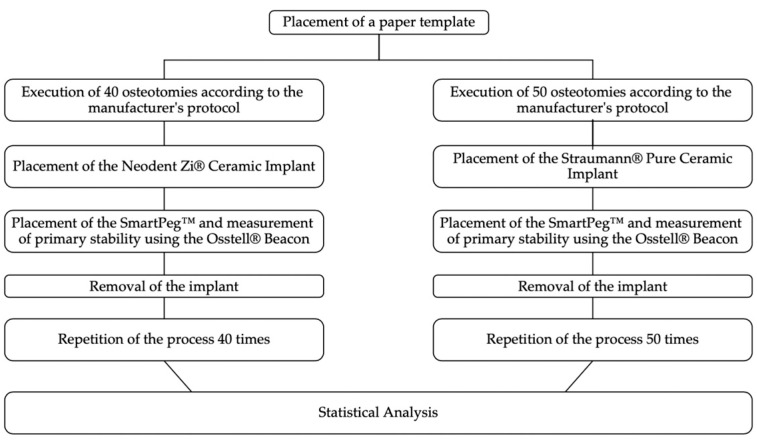
Flowchart representing the experimental procedure.

Prior to preparing the implant site, a paper template was created with the election points for osteotomy, ensuring a distance of at least 3 mm between implants, as recommended by the manufacturer (Figure 3). The entire experimental process is represented in the flow chart in Figure 4.

**Figure 5:**
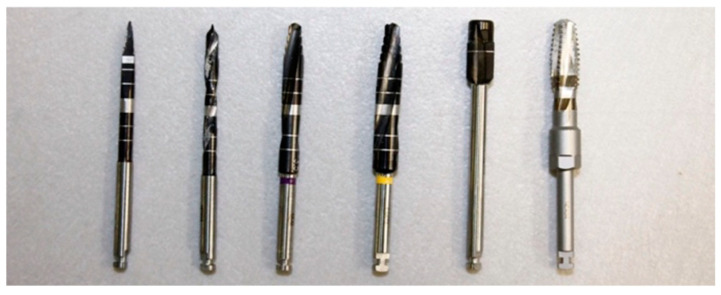
Sequence of drills used, according to the Neodent^®^ protocol.

**Figure 6:**
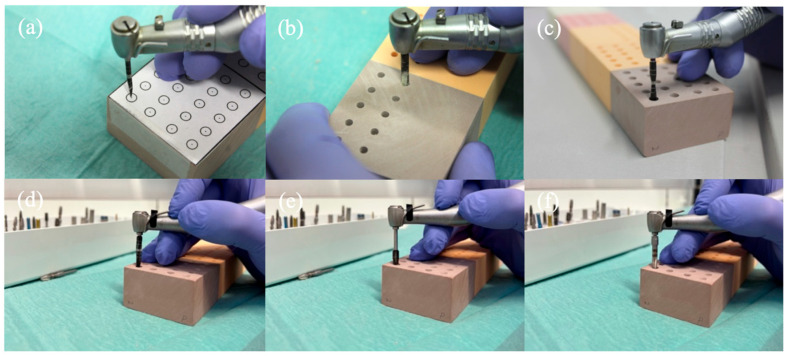
Implant site preparation sequence: (**a**) Initial Needle Drill, (**b**) Tapered Drill Ø 2.0 mm, (**c**) Tapered Drill Ø 3.5 mm, (**d**) Tapered Drill Ø 4.3 mm, (**e**) Countersink Drill Ø 4.3 mm, (**f**) Bone tap Ø 4.3 mm.

**Figure 7:**
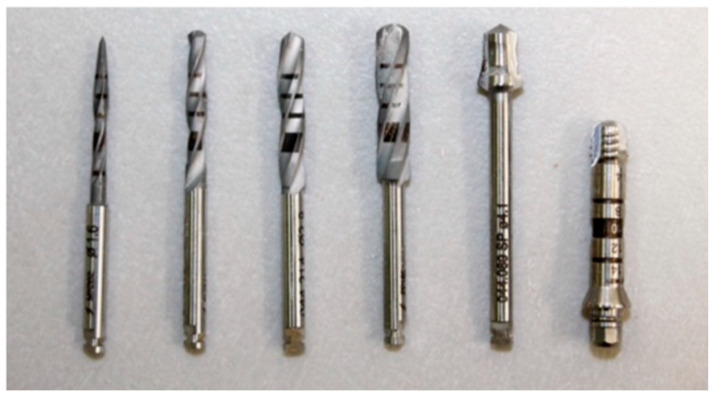
Sequence of drills used, according to the Straumann^®^ protocol.

**Figure 8:**
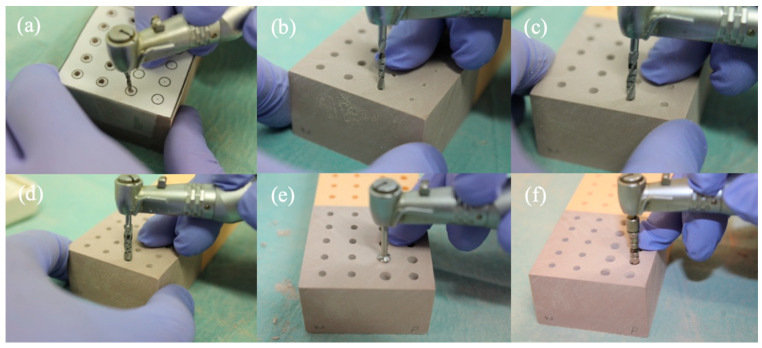
Implant site preparation sequence: (**a**) Needle Drill Ø 1.6 mm, (**b**) Pilot Drill 1 Ø 2.2 mm, (**c**) Pilot Drill 2 Ø 2.8 mm, (**d**) Twist Drill PRO Ø 3.5 mm, (**e**) BL Profile Drill Ø 4.1 mm, (**f**) BL Tap for Adapter Ø 4.1 mm.

**Figure 9:**
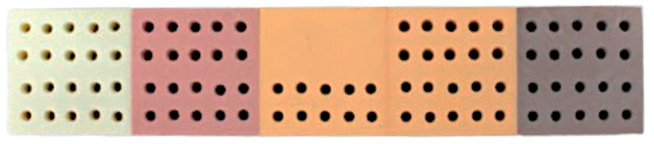
Polyurethane foam block after preparation of the implant sites.

For the placement of the Neodent Zi^®^ Ceramic Implant (Neodent, Curitiba, Brazil), the yellow color-coded surgical kit from Neodent^®^ was used (Figure 5 and Figure 6):

1. Initial Needle Drill (1200 rpm);
2. Tapered Drill Ø 2.0 mm (1200 rpm);
3. Tapered Drill Ø 3.5 mm (1200 rpm);
4. Tapered Drill Ø 4.3 mm (1200 rpm);
5. Countersink Drill Ø 4.3 mm in type III bone (300 rpm);
6. Bone tap Ø 4.3 mm in type I and II bone (30 rpm);

For the placement of the Straumann^®^ Pure Ceramic Implant (Institut Straumann AB, Basel, Switzerland), the surgical kit from Straumann^®^ was used (Figure 7 and Figure 8):

1. Needle Drill Ø 1.6 mm (800 rpm);
2. Pilot Drill 1 Ø 2.2 mm (800 rpm);
3. Pilot Drill 2 Ø 2.8 mm (600 rpm);
4. Twist Drill PRO Ø 3.5 mm (500 rpm);
5. BL Profile Drill Ø 4.1 mm (300 rpm);
6. BL Tap for Adapter Ø 4.1 mm in type I bone (300 rpm);

The polyurethane foam block, shown in Figure 9, displays the arrangement of the implant sites, with the first and second rows corresponding to the osteotomies for the Neodent^®^ Zi Ceramic Implant (Neodent, Curitiba, Brazil) (40 osteotomies), and the third and fourth rows corresponding to the osteotomies for the Straumann^®^ Pure Ceramic Implant (Institut Straumann AG, Basel, Switzerland) (50 osteotomies).

**Figure 10:**
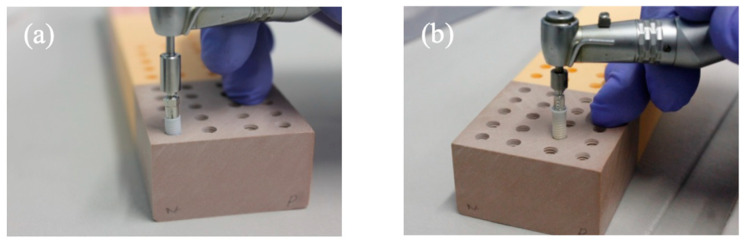
(**a**) Insertion of the Neodent^®^ Zi Ceramic Implant (Neodent, Curitiba, Brazil); (**b**) insertion of the Straumann^®^ Pure Ceramic Implant (Institut Straumann AG, Basel, Switzerland).

After the preparation of the implant sites, the Neodent^®^ Zi Ceramic Implant (Neodent, Curitiba, Brazil) was inserted at a speed of 30 rpm and an insertion torque of 35 N/cm (Figure 10a). The Straumann^®^ Pure Ceramic Implant (Institut Straumann AG, Basel, Switzerland) was inserted at a speed of 15 rpm and an insertion torque of 35 N/cm (Figure 10b). All implant bed preparations and implant placements were performed by final-year students of the Master’s in Dental Medicine course, always under the supervision of an experienced oral surgeon.

### 2.4. Test for Implant Stability

Primary stability was assessed by RFA using the Osstell^®^ Beacon (Osstell, Gothenburg, Sweden).

For the Straumann^®^ Pure Ceramic (Institut Straumann AB, Basel, Switzerland), a SmartPeg™ type 81 (reference 100671) was used, and for the Neodent^®^ Zi Ceramic Implant (Neodent, Curitiba, Brazil), a SmartPeg™ type 99 (reference 100764) was used. The SmartPegs were manually screwed into the corresponding implants. The Osstell^®^ Beacon probe was placed 2–4 mm from the SmartPeg™, without touching it, at an approximate angle of 45°. Three sets of two measurements each were taken, according to the direction (mesio-distal and vestibulo-lingual), and the mean value was used to determine the final ISQ of each tested implant.

After each measurement, each implant was removed using a reverse torque corresponding to the insertion torque.

### 2.5. Statistical Analysis

The statistical analysis of the results obtained using the Osstell^®^ device was conducted with SPSS software (Statistical Package for the Social Sciences v.28, SPSS Inc., Chicago, IL, USA). The aim of the analysis was to compare the implants both between themselves and within each of the five bone densities to determine if their macrogeometry and bone density type had a significant impact on ISQ values. The Shapiro–Wilk test, *t*-tests, the Mann–Whitney U test, or the Kruskal–Wallis test were used according to variable groups, distribution normality, and variance homogeneity. *t*-tests were employed for groups of two qualitative variables with normal distribution, while the Mann–Whitney U test was applied if they had non-normal distribution. The Kruskal–Wallis test was used for groups of three or more variables when the distribution was normal and the variance was non-homogeneous. The level of significance was set at 5% (*p* < 0.05) for all tests performed.

## 3. Results

### 3.1. Evaluation of the Influence of Macrogeometry on Primary Stability

**Figure 11:**
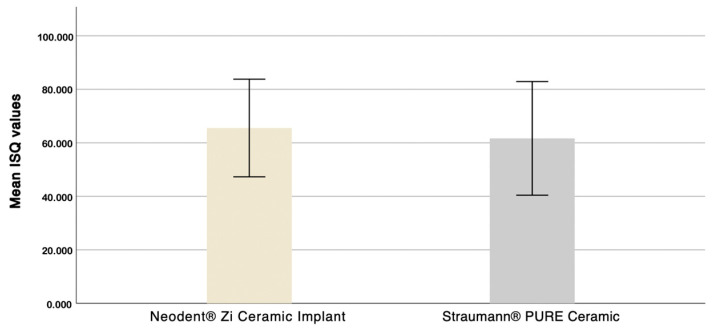
Mean ISQ values distribution.

In this study, a total of 90 implant placements were performed: 40 Neodent^®^ implants and 50 Straumann^®^ implants. The mean ISQ values were 65.54 ± 9.12 for the Neodent^®^ implants and 61.66 ± 10.62 for the Straumann^®^ implants, as shown in Figure 11. Despite the non-normality of the samples, given the large sample size (n ≥ 30), a *t*-test for equality of means was applied. The 95% confidence interval includes zero (−3.319352 to 8.086952), suggesting that while the mean difference is positive, the confidence interval alone is not entirely conclusive. However, based on the mean difference (3.877500 > 0) and the significant *p*-value (*p* = 0.035), we can conclude that the Neodent^®^ Zi Ceramic Implant (Neodent, Curitiba, Brazil) exhibits higher mean ISQ values than the Straumann^®^ Pure Ceramic Implant (Institut Straumann AB, Basel, Switzerland).

**Figure 12:**
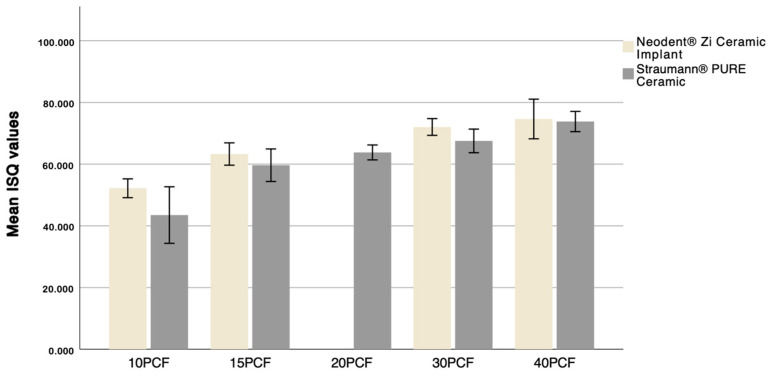
Distribution of mean ISQ values according to implant and bone density.

Table 1 shows the mean ISQ values, standard deviations, and *p*-values for each implant and density. At a density of 10 PCF, the results indicate statistically significant differences in ISQ values between the two implants (*p* < 0.05), with the Neodent^®^ Zi Ceramic Implant (Neodent, Curitiba, Brazil) demonstrating higher stability, as illustrated in Figure 12. For densities of 15 PCF, 30 PCF, and 40 PCF, there are statistically significant differences in ISQ values between the two implants (*p* = 0.001, *p* < 0.05, and *p* = 0.048, respectively), with the Neodent^®^ Zi Ceramic Implant (Neodent, Curitiba, Brazil) performing better.

### 3.2. Evaluation of Straumann^®^ Implant Primary Stability in Different Bone Densities

The results show statistically significant differences in ISQ values across different bone densities (*p* < 0.05). Using the pairwise comparison method (Table 2), it can be concluded that primary stability at 10 PCF is lower than at 20 PCF (*p* < 0.05), 30 PCF (*p* < 0.05), and 40 PCF (*p* < 0.05). Additionally, primary stability at 15 PCF is lower than at 30 PCF (*p* < 0.05) and 40 PCF (*p* < 0.05), and primary stability at 20 PCF is lower than at 40 PCF (*p* < 0.05).

### 3.3. Evaluation of Neodent^®^ Implant Stability in Different Bone Densities

The results indicate statistically significant differences in ISQ values across different bone densities (*p* < 0.05). Using the pairwise comparison method (Table 3), it can be concluded that primary stability at 10 PCF is lower than at 30 PCF (*p* < 0.05), primary stability at 10 PCF is lower than at 40 PCF (*p* < 0.05), and primary stability at 15 PCF is lower than at 40 PCF (*p* < 0.05).

## 4. Discussion

This study aims to understand the specific influence of two different implant macrogeometries—cylindrical and tapered—and the bone density in which they are placed, to inform clinical decisions on implant choice based on bone density.

A polyurethane foam block was used, a material frequently employed in in vitro biomechanical studies in implantology. According to ASTM standards [31], this material is ideal for comparative implant tests due to its uniformity and properties similar to human cancellous bone. Additionally, it minimizes the local and anatomical variability of bone tissue, maintaining mechanical properties consistent with the inorganic component of bone tissue regardless of environmental conditions, and provides a standardized model for dental implant testing [31,32]. However, polyurethane foam does not allow the evaluation of other parameters, such as drilling temperature or histological findings.

Stability measurements were conducted using resonance frequency analysis (RFA), a simple, practical, and reproducible diagnostic method, providing reliable and repeatable implant stability measurements, with the latest Osstell^®^ Beacon device [27]. According to Kittur N et al., RFA is the most reliable method for the in vivo measurement and quantification of implant primary stability [26]. In this study, the Smartpeg™ was manually screwed into place. Although clinicians apply different forces when screwing the transducer, the literature indicates that manual placement allows for objective and reliable RFA measurements [33].

Macrogeometry and the effect of the tapered implants on primary stability have been widely discussed. Compared to parallel-walled implants, the tapered shape may lead to favorable lateral and vertical compression forces during placement and more uniform force distribution, resulting in higher primary stability [23,24]. The results of this study showed statistically significant differences in ISQ values between the different macrogeometries, with the tapered design exhibiting higher ISQ values and thus better primary stability compared to the cylindrical design.

The Neodent^®^ Zi Ceramic Implant (Neodent, Curitiba, Brazil) achieved ISQ values above 70 in the two highest densities (30 PCF and 40 PCF) and values above 60 in the 15 PCF density, which, according to Osstell^®^, indicates high and medium implant stability, respectively [29]. Values below 60 are considered to represent low implant stability, observed mainly in the 10 PCF density of the Neodent^®^ Zi Ceramic Implant (Neodent, Curitiba, Brazil) and in the 10 PCF and 15 PCF densities of the Straumann^®^ Pure Ceramic Implant (Institut Straumann AB, Basel, Switzerland).

The current literature provides limited comparisons between zirconia implant macrogeometries, which limits a comprehensive evaluation of their characteristics and performance compared to other materials. However, the findings of this study are consistent with those of Barikani H et al., who investigated the impact of tapered and cylindrical geometries, as well as the diameter and length of titanium implants, on primary stability using RFA. The ISQ results showed that tapered implants exhibited superior primary stability regardless of their length and diameter [34].

A study by Comuzzi L et al. aimed to evaluate the primary stability of cylindrical and tapered titanium implants positioned in low-density polyurethane foam blocks (10 PCF and 20 PCF), and found that tapered implants showed improved primary stability at 10 PCF density compared to cylindrical microgeometry [35]. In another study, these authors simulated a post-extraction model in polyurethane foam blocks of 10 and 20 PCF, observing consistently higher values for tapered titanium implants under all experimental conditions, suggesting they perform better in artificial post-extraction conditions [36]. These findings are consistent with an in vivo study on the post-extraction sockets of dogs, where a significantly higher average ISQ value was observed for apically tapered titanium implants compared to cylindrical implants immediately after placement [37]. Additionally, animal model studies by García-Vives et al. compared the stability of tapered and cylindrical zirconia implants (10 mm length) in type IV bovine bone, concluding that the tapered shape provides greater primary stability [38].

Similarly, in a prospective clinical study by Lozano-Carrascal et al., insertion torque and ISQ values of 25 cylindrical and 22 tapered titanium implants placed in patients were evaluated, showing that tapered design implants achieved higher ISQ values in both the mandible and maxilla, with values of 71.67 ± 5.16 and 67.2 ± 4.42, respectively [39]. This ISQ difference was attributed by Rokn A et al. to the lateral compressive forces exerted by tapered implants on the surrounding bone walls during placement; they recommended tapered implants in areas with inadequate bone quality and quantity for better primary stability [40].

On the contrary, some studies present conflicting results. A study by Heitzer M et al. compared tapered titanium and cylindrical zirconia implants (Straumann^®^ Pure Ceramic) placed in the anterior maxillary bone of cadavers under low-bone-density criteria and in 10 PCF and 20 PCF polyurethane foam blocks. They found significantly higher mean ISQ values for the cylindrical implants compared to the tapered titanium implants in both the cadaver and the polyurethane sponge blocks, suggesting that cylindrical zirconia implants are a viable treatment option comparable to conical titanium implants in low-density human bone [41].

These results also differ from a study by Bilhan et al., conducted on an animal model, where cylindrical titanium implants exhibited higher ISQ values compared to tapered implants. The authors explained these differences by noting that tapered implants were placed in cancellous bone, with primary stability entirely dependent on the cervical portion, increasing the risk of losing stability potentially obtained from the apical half, thus presenting a disadvantage for tapered implants [42].

However, a meta-analysis showed that, based on the available literature, the ISQ values of tapered titanium implants measured immediately after placement did not show statistically significant differences compared to cylindrical implants. Therefore, RCTs are needed to validate the use of tapered implants in immediate loading protocols and other complex clinical scenarios [43].

Addressing the influence of bone density on implant primary stability through ISQ values, statistically significant differences were found, indicating that bone density impacts primary stability for both implant macrogeometries. Results suggest that the highest ISQ values were achieved at 40 PCF density, corresponding to the highest density studied, for both the Neodent^®^ Zi Ceramic Implant (Neodent, Curitiba, Brazil) and the Straumann^®^ Pure Ceramic Implant (Institut Straumann AG, Basel, Switzerland). It was also observed that, on average, ISQ values increased with density, concluding that increased density leads to increased primary stability, regardless of implant macrogeometry.

Ivanova et al. conducted an in vivo study to evaluate the influence of various factors on implant primary stability, including bone density. The study inserted 90 implants into the maxilla and mandible of different patients, showing that primary stability values increased proportionally with bone density, concluding that bone density positively influences primary stability acquisition [44].

This study has some limitations, including the high number of consecutive insertions and removals of the two implants, the reuse of the SmartPeg™, the use of polyurethane foam blocks without cortical bone, and the differences in the length and diameter of the implants.

Notably, the high number of consecutive insertions and removals of the implants could potentially distort the macrogeometry of the threads, thereby introducing uncertainty in the ISQ results. This choice can be justified by financial and logistical constraints associated with studies in oral implantology. We recommend the use of different implants in each measurement in future studies to eliminate this bias.

Additionally, only one SmartPeg™ per implant was used, and its reuse might have led to gradual wear of the aluminum structure or a less precise fit to the implant, resulting in inaccurate ISQ measurements. Clinically, this could cause prosthetic complications due to aluminum particle release, preventing precise adaptation and stability of the abutment to the implant [45].

Furthermore, osteotomies in this study were performed without irrigation. However, according to Di Stefano et al., irrigation in polyurethane foam blocks has no impact on primary stability parameters using RFA [46].

An in vitro study by Chávarri-Prado et al. investigated the influence of bone cortical on primary stability, using two polyurethane blocks, one with and one without a cortical layer, concluding that cortical presence significantly affects primary stability values measured by ISQ. Therefore, using a test body imitating bone cortical in this study would have allowed for a more accurate simulation of the oral cavity [47].

Another consideration potentially affecting results is the inexperience of the clinician. The literature indicates that as clinicians gain experience and practice, they improve their techniques and approach, contributing to superior clinical outcomes, higher success rates, and fewer complications [48].

Finally, the differences in the length and diameter of the implants used in this study may alter the results. The published literature on this relationship is controversial. Previous investigations have shown that longer implants achieve lower primary stability [49], while others suggest that primary stability increases with length [50], or that implant length and diameter do not influence primary stability [40,51]. The perception that larger and longer implants have greater stability may not be accurate. Implant design, thread geometry, and surrounding bone may have a more significant effect on mechanical stability.

Future research should explore different study designs to validate these findings, including in vivo studies, animal studies, and randomized clinical trials in patients, as these allow for the analysis of variables that are difficult to study under in vitro conditions, such as tissue response to the implant, bone formation around the implant, and the impact of factors like bone remodeling and healing. Additionally, studies with larger sample sizes and variables, including different bone densities and types of implants (ceramic and titanium), could provide more reliable data. Comparing the performance of implants made from different materials, with varying sizes and modified surfaces, would help clarify the efficacy of each in specific situations, such as in areas of low bone density.

## 5. Conclusions

Within the limitations of this in vitro study, the following conclusions were drawn:

▪ The macrogeometry of zirconia dental implants influences primary stability values.
▪ The tapered implant demonstrated superior ISQ results compared to the cylindrical design in every tested bone density.
▪ Higher primary stability values are associated with increased bone density.

Therefore, the results suggest that, in clinical situations with higher bone density, both types of implants may provide sufficient stability. However, in cases of low bone density, the choice of a conical implant may enhance initial stability. This is particularly relevant in areas such as the posterior maxilla, where bone density is often lower, and achieving primary stability can be more challenging. In such cases, the use of a rough and self-tapping titanium implant could further improve primary stability and be a recommended option. These findings may assist dental professionals in making more informed decisions when selecting implant designs, considering the patient’s bone density. Customizing treatment based on these variables can improve clinical outcomes and reduce the risk of implant failure, thereby increasing the predictability of results across different types of bone.

Regarding future research, further validation of these findings could be achieved through in vivo studies, animal models, and randomized clinical trials.

## Figures and Tables

**Table 1:** Mean ISQ values, standard deviations, and *p*-values for each implant and bone density.

Densities	Mean ± SD		*p*-Value
	Neodent^®^	Straumann^®^	
10 PCF	52.18 ± 1.52	43.5 ± 4.58	<0.001
15 PCF	63.28 ± 1.81	59.65 ± 2.64	0.001
20 PCF		63.80 ± 1.21	
30 PCF	72.05 ± 1.36	67.53 ± 1.91	<0.001
40 PCF	74.63 ± 3.21	73.82 ± 1.64	0.048

**Table 2:** Pairwise comparison method for evaluation of primary stability of Straumann^®^ Pure Ceramic Implant (Institut Straumann AB, Basel, Switzerland) across different bone densities.

Comparisons	Sig.	Adj. Sig. *
10 PCF–15 PCF	0.104	1.000
10 PCF–20 PCF	0.002	0.024
10 PCF–30 PCF	<0.001	0.000
10 PCF–40 PCF	<0.001	0.000
15 PCF–20 PCF	0.158	1.000
15 PCF–30 PCF	0.004	0.036
15 PCF–40 PCF	<0.001	0.000
20 PCF–30 PCF	0.133	1.000
20 PCF–40 PCF	0.002	0.019
30 PCF–40 PCF	0.111	1.000

* The significance values were adjusted using Bonferroni correction for multiple tests.

**Table 3:** Pairwise comparison method for evaluation of primary stability of Neodent^®^ Zi Ceramic Implant (Neodent, Curitiba, Brazil) across different bone densities.

Comparisons	Sig.	Adj. Sig. *
10 PCF–15 PCF	0.056	0.334
10 PCF–30 PCF	<0.001	0.000
10 PCF–40 PCF	<0.001	0.000
15 PCF–30 PCF	0.020	0.118
15 PCF–40 PCF	<0.001	0.004
30 PCF–40 PCF	0.284	1.000

* The significance values were adjusted using Bonferroni correction for multiple tests.

## Data Availability

The data that support the findings of this study are available from the corresponding author upon reasonable request.
